# Sinus Bradycardia Associated with Remdesivir Treatment in COVID-19: A Case Report and Literature Review

**DOI:** 10.3390/jcdd8020018

**Published:** 2021-02-12

**Authors:** Fotios Barkas, Chrysoula-Paraskevi Styla, Aris Bechlioulis, Haralampos Milionis, Evangelos Liberopoulos

**Affiliations:** 1 Department of Internal Medicine, University Hospital of Ioannina, 45110 Ioannina, Greece; fotisbarkas@windowslive.com (F.B.); chrisapst@gmail.com (C.-P.S.); hmilioni@uoi.gr (H.M.); 2 Department of Cardiology, University Hospital of Ioannina, 45110 Ioannina, Greece; md02798@yahoo.gr

**Keywords:** SARS-CoV-2, COVID-19, remdesivir, bradycardia, arrhythmia

## Abstract

Background: Although remdesivir treatment is widely used during the pandemic coronavirus disease 2019 (COVID-19), there is scarce evidence regarding its cardiac side effects. Case presentation: We report the case of a 36-year-old male hospitalized due to severe COVID-19 symptoms. He presented with a 10-day history of fever (up to 39.7 °C), productive cough, hemoptysis, fatigue, myalgias and hypoxemia. The patient received supplemental oxygen, dexamethasone, remdesivir and empirical antibiotic treatment according to protocol. Asymptomatic sinus bradycardia developed on hospital day 3 (namely, heart rate 39/min compared to 92/min on admission). Secondary causes of bradycardia were excluded based on the absence of relevant evidence from laboratory work-up and echocardiographic examination. The patient’s rhythm restored to normal 9 days after the discontinuation of remdesivir. Conclusions: Considering the frequent use of remdesivir in patients with COVID-19, physicians should be aware of this possible adverse event.

## 1. Introduction

At the end of 2019, a novel coronavirus, severe acute respiratory syndrome coronavirus 2 (SARS-CoV-2), was identified as the cause of a cluster of pneumonia cases in Wuhan, in the Hubei Province of China and finally declared a pandemic in February 2020 [1]. Until early 2021, a total of 92.3 million coronavirus disease 2019 (COVID-19) cases and 1.98 million deaths have been confirmed worldwide [1]. Although remdesivir, an antiviral drug with in-vitro inhibitory effect against SARS-CoV-2, has been proposed for the treatment of patients infected with SARS-CoV-2 and widely used during the pandemic COVID-19 [2,3], there is scarce evidence regarding its cardiac side effects [2,4,5,6].

## 2. Case Presentation

A 36-year-old male presented with a 10-day history of fever up to 39.7 °C, productive cough with hemoptysis, fatigue and myalgias. Polymerase chain reaction (PCR) testing of a nasopharyngeal sample confirmed SARS-CoV-2 infection, and the patient was admitted to the Infectious Disease Unit of the University Hospital of Ioannina, Greece.

On admission, the patient was febrile (body temperature 38.5 °C) but hemodynamically stable (blood pressure 113/72 mmHg). Analysis of arterial blood gases showed hypoxia (oxygen saturation; SpO_2_: 88%, partial pressure of oxygen; PO_2:_ 59.3 mmHg) while breathing room air. He was put on supplemental oxygen (nasal cannula 4 L/min, FiO_2_ = 36%). An electrocardiogram (ECG) revealed normal sinus rhythm (heart rate 92/min). Body mass index was 35.3 kg/m^2^. Laboratory tests are shown in Table 1. A chest x-ray showed bilateral middle and lower zone consolidation opacities peripherally distributed. A chest CT scan revealed lung abnormalities compatible with severe coronavirus disease 2019 (COVID-19), including “crazy paving” and ground-glass opacities bilaterally, mainly of the lower lobes, occupying 50–75% of the lung parenchyma.

The patient was treated according to protocol with antibiotic therapy (ceftriaxone 2 g qd and vibramycin 100 mg bid), dexamethasone 6 mg qd, remdesivir (a loading dose 200 mg on day 1 followed by 100 mg qd), and low molecular weight heparin for thromboprophylaxis (enoxaparin 60 mg bid).

**Figure 1:**
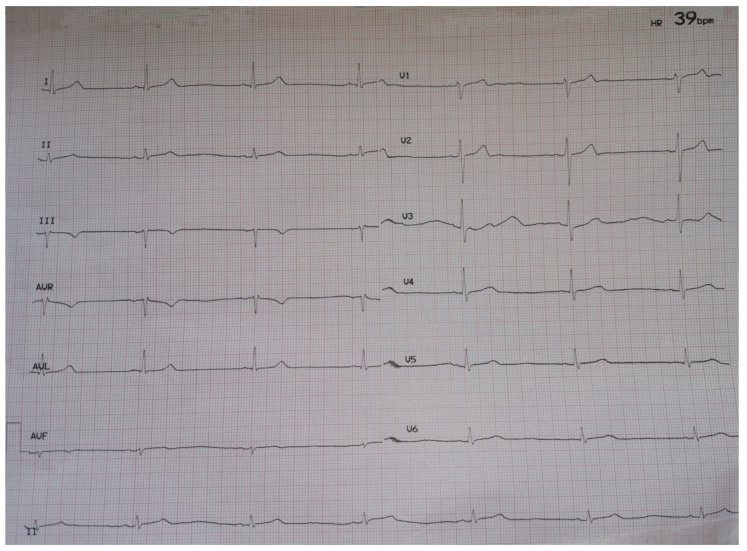
ECG on hospital day 3.

On hospital day 3, there was evidence of asymptomatic sinus bradycardia (39 bpm) (Figure 1). The patient remained hemodynamically stable (blood pressure 121/70 mmHg), afebrile, while PO_2_ was 89 mmHg on oxygen supplementation (FiO_2_ = 36%).

**Figure 2:**
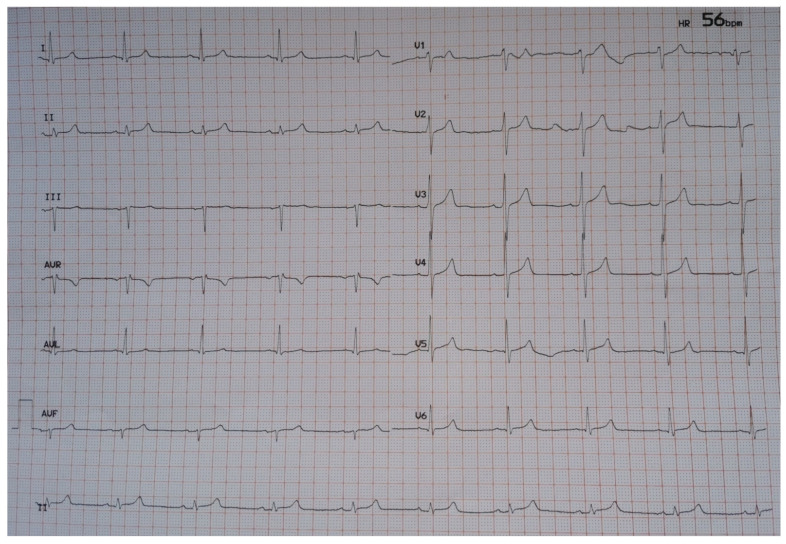
ECG at discharge (hospital day 12).

Physical examination was unrevealing while extensive laboratory evaluation and echocardiogram was not indicative of secondary causes of sinus bradycardia, such as myocardial ischemia, myocarditis, thyroid dysfunction, electrolyte disorders or massive pulmonary embolism. Sinus bradycardia was deemed to be related with remdesivir treatment, which was accordingly discontinued. Heart rate gradually restored to normal. On hospital day 12, the patient required no supplemental oxygen and an ECG showed sinus rhythm with a heart rate of 58/min (Figure 2).

## 3. Discussion

Herein, we report a case of possible remdesivir-associated sinus bradycardia in a patient with severe COVID-19. To the best of our knowledge, there is a handful of reports in the literature, with ours being the third case report.

SARS-CoV-2 has been associated with cardiovascular complications, including myocardial infarction, myocarditis and rhythm abnormalities [7,8]. Arrhythmias include supraventricular tachycardia, atrial fibrillation, atrial flutter, complete heart block, cardiac arrest, as well as polymorphic, monomorphic and multifocal ventricular tachycardia [9]. Of note, transient sinus bradycardia has been reported in four critically ill patients with COVID-19 [10]. All patients were febrile and intubated, whereas two of them had established cardiovascular disease [10]. After excluding secondary causes of sinus bradycardia, the authors concluded that relative bradycardia could be a possible manifestation of COVID-19 [10]. Remdesivir was not administered in any of those patients [10]. Severe hypoxia, inflammatory damage of cardiac pacemaker cells in the setting of myocarditis, myocardial ischemia and strain, electrolyte disturbances, intravascular volume imbalances and drug side effects could be plausible causes of arrhythmias in patients with COVID-19 [9,10]. Moreover, increased cytokine production might exhibit an inhibitory effect on the sinoatrial node [9,10]. It could be argued that bradycardia could be a COVID-19-related symptom in our patient. In contrast to the previously described cases, our patient neither was critically ill requiring invasive ventilation and/or inotropes nor was diagnosed with cytokine release syndrome. In addition, he had no signs of relative chronotropic incompetence upon his admission, when he was febrile and hypoxemic.

Remdesivir, a nucleoside analogue prodrug acting as an RNA polymerase inhibitor, has been shown to shorten the time to recovery in hospitalized adults with severe COVID-19 requiring low-flow supplemental oxygen [2,3]. Frequent side effects include hepatotoxicity, gastrointestinal symptoms, and nephrotoxicity [11]. Limited information exists regarding any potential cardiac side effects associated with its use. Hypertension, cardiac arrest and atrial fibrillation have been reported in a few cases of COVID-19 patients treated with remdesivir; however, these conditions were not confirmed in randomized placebo-controlled trials [2,6]. The U.S. Food and Drug Administration does not file sinus bradycardia as an adverse effect or warning in the factsheet of emergency use authorization of remdesivir for COVID-19 [12].

Our case adds to the scarce evidence of remdesivir therapy related with sinus bradycardia [4,5]. Three patients (26–77 years old) with moderate/severe COVID-19 have been reported with sinus bradycardia within 3 days after remdesivir initiation [4,5]. Similar to our case, those patients had no previous cardiovascular disease, did not require invasive ventilation, other secondary causes were excluded, and bradycardia resolved after the discontinuation of the culprit drug [4,5]. The potential drug-induced mitochondrial dysfunction caused by the strong affinity of remdesivir for human mitochondrial RNA polymerase (h-mtRNAP) and atrioventricular nodal inhibition due to its resemblance with adenosine have been proposed as potential mechanisms [4]. Considering that mitochondrial dysfunction from other drugs is a well-known cause of cardiotoxicity, the possibility of h-mtRNAP involvement and subsequent mitochondrial dysfunction might be plausible in the case of remdesivir [4]. In addition, given its resemblance with adenosine triphosphate, remdesivir could inhibit atrioventricular node by binding to the A1 receptor on cardiac cells [4]. Of note, our patient suffered from sinus, rather than atrioventricular node, dysfunction. Despite the theoretical similarity of remdesivir to adenosine triphosphate potentially affecting the atrioventricular node, all published cases have reported sinus bradycardia. Therefore, another pathophysiological mechanism probably exists. Whether a follow-up of these patients for future symptomatic sinus node disease may be needed is currently unknown.

## 4. Conclusions

Taking into account the incorporation of remdesivir in treatment protocols of COVID-19, clinicians, who are currently watching with a vigilant eye for cardiac rhythm abnormalities in these patients, should be aware of the reversible association of remdesivir with sinus bradycardia.

## Figures and Tables

**Table 1:** Laboratory tests on hospital days 1 and 3.

	Day 1	Day 3	Reference Range
Hematocrit (%)	41.6	41	41–53
Hemoglobin (g/dL)	14.3	13.8	13.5–17.5
White cell count (per μL)	2410	5930	4500–11,000
Absolute neutrophil count (per μL)	1614	4625	1500–8000
Absolute lymphocyte count (per μL)	660	1000	1000–4800
Platelet count (per μL)	119,000	168,000	150,000–400,000
International normalized ratio	0.98	-	≤1.1
D-dimers (μg/mL)	0.56	0.49	<0.5
Fasting plasma glucose (mg/dL)	120	93	70–125
Creatinine (mg/dL)	0.76	0.64	0.6–1.2
Potassium (mmol/L)	4.84	4.41	3.5–5.3
Sodium (mmol/L)	137	140	136–146
Chloride (mmol/L)	102	104	98–106
Magnesium (mmol/L)	1.78	-	1.3–2.1
Alanine aminotransferase (U/L)	24	117	10–35
Aspartate aminotransferase (U/L)	38	59	10–35
Lactate dehydrogenase (U/L)	494	253	115–230
Creatinine kinase (U/L)	52	17	25–160
High-sensitivity troponin (pg/mL)	5.5	4.0	0.0–11.6
Ferritin (mg/dL)	708	-	11.0–306.8
C-reactive protein (mg/L)	86	12	<6
Thyroid stimulating hormone (μU/mL)	0.33	-	0.38–5.33
Interleukin-6 (pg/mL)	2.5	-	0.0–6.4
pH	7.47	7.44	7.36–7.44
PO_2_ (mmHg)	59.3	89	≥60
PCO_2_ (mmHg)	33.9	37.3	36–44
HCO_3_ (mEq/L)	24.1	24.8	21–27
Anion gap (mmol/L)	11.3	12.2	3–9
Lactate (mmol/L)	1.0	1.4	0.4–2.0
FiO_2_ (%)	21	36	-

## Data Availability

Data available on request due to restrictions of privacy.
